# Unbiased Decisions Among Women’s Basketball Referees

**DOI:** 10.3389/fpsyg.2020.566684

**Published:** 2020-11-05

**Authors:** Carlos Gomez-Gonzalez, Helmut Dietl, Cornel Nesseler

**Affiliations:** ^1^Department of Business Administration, University of Zurich, Zurich, Switzerland; ^2^Norwegian University of Science and Technology (NTNU) Business School, Norwegian University of Science and Technology, Trondheim, Norway

**Keywords:** bias, gender, nationality, race, referees, split-second decision

## Abstract

Decisions often reflect implicit biases. Ethnic, racial, and gender traits are associated with stereotypes that may influence the decision-making process. Previous research shows that referees’ decisions in men’s professional sports are often biased in favor of racial and nationalistic in-groups. This study examined if similar biases exist in women’s professional sports. Additionally, this study analyzed the potential influence of the gender composition of referee teams on rapid decisions. We gathered data on referee foul calls in women’s professional basketball in Spain, 2014–2019 and defined important decisions (fifth fouls) and stressful situations (one-possession matches). The main finding is that out-groups based on racial (i.e., Black players) and nationalistic (i.e., foreign players) criteria did not differ in number of foul calls received. In stressful situations, foreign players actually received fewer fouls than Spanish players. Similarly, there was no evidence of bias due to the gender composition of referee teams: foul calls did not differ between all-male and mixed teams. Implications for race and nationality as dynamic social constructs within ethnocentric and social identity theories are discussed.

## Introduction

Research demonstrates that decisions and evaluations often hide an implicit bias, even if we do not intend it or realize it (Staats, 2014). The Implicit Association Test has been used to capture the extent to which social groups are implicitly associated with good/bad traits (Greenwald et al., 1998). This type of association is subconscious and probably a result of early exposure to cultural beliefs and stereotypes. Banaji and Greenwald (2016) call it the “blindspot.” Implicit biases tend to result in discriminatory behaviors that favor in-group members (Staats, 2014).

Implicit bias is, therefore, an alternative explanation to traditional taste-based and statistical discrimination (Bertrand et al., 2005). Multiple studies find evidence that implicit racial/ethnic biases influence the evaluation of police officers and judges (Banks et al., 2006), doctors and health care professionals (FitzGerald and Hurst, 2017), employment recruiters (Dipboye and Colella, 2013), policy makers (Glaser et al., 2014), students and voters (Jost et al., 2009), and sport referees (Price and Wolfers, 2010). Most studies use data from the United States In sports, most studies on biases among referees use data from men’s competitions. Still, ethnicity, nationality, and race are dynamic social constructs that are likely to change over time and develop differently across societies. More research is needed to understand racial and nationalistic biases in different countries and alternative settings, such as women’s sports.

Research has also shown that the widespread barriers and biases against racial and ethnic minorities can be implicit (unconscious) or explicit (conscious) (Blair et al., 2015). The analysis of referee decisions in sports is especially relevant to the literature on biases (Dohmen and Sauermann, 2016), because calling a foul is a split-second decision, and usually made under pressure, when implicit association biases should matter the most. Any unconscious association of racial/ethnic groups with negative traits is likely to surface in this situation and bias the decisions of referees.

Another factor that may influence these decisions is the gender composition of referee teams. The representation of women in sports is limited, especially in leadership positions (Kane and Stangl, 1991; Walker and Bopp, 2011). Women are also underrepresented on referee teams. In our sample of women’s basketball games in Spain, we found only 25 female referees (approximately 9% of the total pool of referees). Despite this underrepresentation, we can test if the gender composition of referee teams has an influence on the number of fouls (approximately 18% of the matches have at least one woman as a referee). The relationship between gender diversity and team performance is widely analyzed in sports (Lee and Cunningham, 2019) and other domains (Azmat, 2019). However, the decision-making of referee teams as a function of gender composition has been overlooked to date.

In this paper, we analyze professional women’s basketball in Spain; the main focus is on referee decisions that may reflect an implicit bias against out-group players based on race/ethnicity and nationality. The sample includes 53,398 player-match observations. In addition, we examine the influence of gender composition of referee teams on foul calls. The dataset allows us to control for a number of factors regarding player and team characteristics that may moderate the role of racial/ethnic and nationalistic biases. Moreover, we extend the analysis to test for biases in important calls (i.e., the fifth foul, which disqualifies a player for the rest of the match) and stressful situations (i.e., one-possession matches, where the last possession decides the winner).

The article is organized as follows: the second section reviews the related literature, shapes the theoretical contribution, and provides the hypotheses. The third section describes the data and explains the empirical strategy, and the fourth section presents the results and discusses the implications for research. Conclusions are drawn in the fifth section.

## Background and Hypotheses

### Biases in Referee Decisions

Referees are an ideal group of decision makers for research purposes. Their decisions are observable and important for the outcome of the match. They frequently make match-changing decisions: calling or not calling a foul may change the outcome of a match and may even decide championships, which, in turn, have significant economic consequences. Therefore, not only the performance of athletes but also the decisions of referees are constantly under scrutiny.

Additionally, referees often have to make decisions in a split second. Calling or not calling a foul in a fast-paced sport like ice hockey or basketball is a decision made within a tenth of a second. Therefore, such decisions cannot be driven by strategic contemplation. A foul must be called immediately, leaving no time for elaborate conscious deliberation.

The ideal referee makes unbiased decisions and treats all athletes equally regardless of their race, nationality, gender, sexual orientation, or religion. In fact, referees receive specific education and training to apply the laws of the game consistently and fairly. A sport loses its integrity when its referees consciously favor one type of athlete over another. But even if referees do not discriminate against certain kinds of athletes consciously, they may still do so sub- or unconsciously; that is, referee decisions may be subject to implicit biases (cf. Greenwald and Krieger, 2006).

Findings on racial, ethnic, and nationalistic biases are present across fields, occupations, and cultures (Jost et al., 2009). Research has extensively examined the determinants of ethnic, racial, and national identity that are used to create in-group and out-group distinctions (Tajfel, 1982). In-group favoritism often occurs, even when there is no personal advantage. The process of social categorization explains some of the mechanisms behind in-group preferences (van Knippenberg et al., 2004). The creation of social groups and the categorization of their members lead to stereotypes that may result in racial and nationalistic implicit biases.

Race represents a powerful social construct of interest to research (Gannon, 2016). The social meaning of race has historically allowed cultures to create in-group and out-group labels, determining relationships, attitudes, and social status (Park, 1950). Similarly, nationality is an important source of social identity that determines several aspects of human experience (Rad and Ginges, 2018) and interactions (Fox and Miller-Idriss, 2008).

In sports, bias among referees and judges has attracted research attention (Dohmen and Sauermann, 2016). The seminal paper of Price and Wolfers (2010) on biases among National Basketball Association (NBA) referees set the ground for several contributions that followed. The authors analyzed the racial composition of referee teams and the number of fouls called on opposite-identity players. They found a significant bias: players received up to 4% fewer fouls calls when referees belonged to the same racial group. Similarly, Parsons et al. (2011) found that when Major League Baseball umpires and batters shared ethnicity/race, the probability of a called strike was lower. Based on the existing literature on racial biases among sport referees, we make the following prediction:

Hypothesis 1: Black players are called for more fouls than their white counterparts.

Beyond racial biases in the United States, there is also evidence of nationalistic preferences of referees and judges in different sports and countries. Gallo et al. (2013) found that referees in the English Premier League who were born and spent their entire lives in the United Kingdom were 15% more likely to punish foreign, non-white players with yellow cards. Pope and Pope (2015) found that Union of European Football Associations (UEFA) Champions League football referees tend to favor players from the same country.

Other researchers report nationalistic biases when referees award free kicks in Australian football (Mohr and Larsen, 1998) and penalties in international rugby competitions (Page and Page, 2010). Research has also found nationalistic preferences in the evaluations of ski jumpers, figure skaters (Zitzewitz, 2006, 2014; Krumer et al., 2020), and dressage riders (Sandberg, 2018). Based on the existing literature on nationalistic biases among referees, we make the following prediction:

Hypothesis 2: Foreign players are called for more fouls than their national counterparts.

### Gender Composition of Referee Teams

Personality traits and risk preferences of men and women are often used to explain differences in sports outcomes at the individual level. For example, in judo, men benefit from psychological momentum, but women do not (Cohen-Zada et al., 2017b). Also, whereas Cohen-Zada et al. (2017a) found that in tennis men are more likely to choke under pressure, Lindner (2017), observing biathlon, found no gender differences.

Research also examines the influence of gender diversity on group performance (e.g., Wicker et al., 2012). Lee and Cunningham (2019) review the relationship between gender diversity and sport outcomes, and find an overall positive effect. However, several studies provide mixed evidence, and a number of moderating factors have been identified; namely, the type of setting, outcome, and sport role (e.g., administrators, coaches, or players). Regarding sport role, referees have been largely overlooked.

Souchon et al. (2004) examined penalties called by male referees on male and female handball players. The authors found that female players were more likely to receive sanctions than their male counterparts. In a study analyzing videos of referees’ decisions, Souchon et al. (2013) explored the influence of gender on the evaluation of penalties against male and female players and did not find significant differences. To the best of our knowledge, no previous research has considered implicit biases in professional sports as a function of the gender composition of referee teams.

Documented differences in decisions lead to disparate outcomes for women and men in the workplace (Chang and Milkman, 2020). Both men and women exhibit gender biases that tend to punish women, especially for displaying stereotypical male behaviors. However, there is scant evidence on the possible influence of group gender composition on decisions, despite its relevance to the decision-making process.

Regarding risk preferences, evidence from laboratory experiments is mixed. Booth et al. (2014) found that women were more likely to take risks in all-female groups than in mixed groups, whereas Castillo et al. (2019) found that women become more risk tolerant as the proportion of men increases. In natural settings, studies also find inconsistent results. Bagues et al. (2017) reported that the presence of women evaluators on a committee does not alter the quantity or quality of selected candidates to professorships in Italy and Spain. De Paola and Scoppa (2015), however, found that all-male science committees, unlike mixed-gender ones, are biased against women candidates.

These studies theorize that the presence of women could induce a “licensing effect” (Monin and Miller, 2001), such that with women present, men may feel licensed to behave differently, or male identities may be strengthened, which in turn might alter the outcome (Akerlof and Kranton, 2000). In basketball, toughness and aggressiveness are stereotypical male behavior, which, according to the licensing effect, may lead mixed referee teams to behave differently than all-male teams. Based on the existing literature on the licensing effect, we make the following prediction:

Hypothesis 3: The number of foul calls differs between all-male and mixed referee teams.

## Data and Methods

### Data Description

We gathered data on players, coaches, and referees for the women’s first division (765 matches) and second division (1,790 matches) in Spain. The database covers five seasons (2014–2015 to 2018–2019) and 58 teams.

In total, the database has 53,398 observations at the player-match level. We used the official websites of the leagues^1^ to gather match statistics, as well as information on teams, coaches, players, and referees. The race of players was inferred by looking at pictures in newspapers and on league and team websites. Table 1 displays the complete list of summary statistics.

**TABLE 1:** Summary statistics.

	All foreign		Foreign black		Foreign white		Spanish	
				
	Mean	SD	Mean	SD	Mean	SD	Mean	SD
**Raw player statistics**								
Minutes played	25.18	9.15	25.73	9.05	20.98	10.08	20.25	10.09
Points	9.81	6.66	10.73	6.84	6.45	5.80	5.97	5.56
Fouls	2.15	1.37	2.20	1.34	1.83	1.38	1.77	1.37
Player productivity – Stats*40/Min played								
Fouls	3.86	3.57	3.90	3.47	4.00	4.38	4.04	4.51
Points	14.69	8.67	15.89	8.72	11.05	9.00	10.57	9.03
Free throws made	2.46	2.95	2.79	3.08	1.79	3.11	1.73	3.17
Free throws missed	1.08	1.83	1.30	1.91	0.91	2.23	0.92	2.32
2 point goals made	4.69	3.49	5.45	3.59	3.03	3.25	2.86	3.23
2 point goals missed	5.60	3.98	6.31	4.09	4.29	4.02	4.17	4.06
3 point goals made	0.95	1.62	0.73	1.42	1.06	1.86	1.04	1.87
3 point goals missed	2.17	2.71	1.76	2.37	2.75	3.48	2.78	3.57
Offensive rebounds	2.59	2.90	3.03	3.17	1.78	2.69	1.70	2.72
Defensive rebounds	5.63	4.14	6.11	4.27	4.31	4.02	4.14	4.01
Assists	2.01	2.28	1.92	2.23	2.14	2.79	2.16	2.87
Steals	1.61	1.95	1.72	2.03	1.59	2.30	1.61	2.38
Blocks	0.53	1.21	0.63	1.33	0.31	1.03	0.28	1.02
Turnovers	3.23	3.12	3.39	3.07	3.10	3.59	3.11	3.67
**Player information**								
Black player	0.50	0.50	-	-	-	-	0.02	0.14
International	0.52	0.50	0.44	0.50	0.14	0.35	0.05	0.22
Top 20 ranked nation	0.17	0.38	0.11	0.31	0.08	0.27	0.05	0.22
Top 40 ranked nation	0.19	0.39	0.15	0.36	0.04	0.19	-	-
Top 60 ranked nation	0.11	0.32	0.15	0.36	0.01	0.11	-	-
Top 80 ranked nation	0.04	0.20	0.02	0.15	0.01	0.10	-	-
Age	26.15	3.93	25.82	3.97	24.49	4.92	24.10	5.02
Player drafted	0.10	0.29	0.14	0.35	0.01	0.09	0.00	0.03
Center	0.20	0.40	0.24	0.43	0.14	0.35	0.14	0.35
Guard	0.30	0.46	0.29	0.45	0.31	0.46	0.31	0.46
Power forward	0.18	0.39	0.17	0.37	0.15	0.36	0.14	0.35
Shooting guard	0.07	0.26	0.09	0.28	0.14	0.35	0.16	0.36
Small forward	0.24	0.43	0.22	0.41	0.23	0.42	0.23	0.42
**Referees**								
All-male	0.82	0.38	0.82	0.38	0.83	0.38	0.83	0.38
Mixed	0.16	0.37	0.16	0.37	0.16	0.36	0.16	0.36
All-female	0.02	0.13	0.02	0.12	0.02	0.12	0.01	0.12
**Sample information**								
Players	450		229		221		1004	
Total minutes	335,790		170,408		165,382		690,433	
Referees	288							
Matches	2,555							
Division 1 match	765							
Derby match	254							
Regular season	2,448							
Seasons	5							

Players’ statistics included the number of fouls per match, minutes played, points, and shooting efficiency. Players’ age and status (drafted and/or international) were also gathered. Drafted and international players might have not only better skills, but also a “superstar” reputation that may bias the decisions of referees. “Drafted” refers to players selected by the Women’s National Basketball Association (WNBA), the top women’s basketball league in the United States International players refer to players that compete with their respective national teams. Finally, we included player’s position, which is expected to influence the number of fouls (Sampaio et al., 2006).

Several teams in the Spanish league are located in the same city or close to each other. We controlled for a potential derby effect with a binary variable that classified matches as a derby if the teams operate within a 50-km radius of each other (cf. Buraimo et al., 2012). At the end of the season, the best six teams qualified for the playoffs. The top two teams directly qualified for one semifinal. The other four teams compete in best-of-three quarterfinals. We controlled for a potential playoff effect with a binary variable that classified matches as regular season vs. playoff (Price et al., 2012).

Referee teams for the dataset were composed of two referees with one exception. In the 2018–2019 season, Division 1 matches had three referees. We dropped this season from the sample, so that the variable that captures gender composition of referee teams is homogeneous. (The results remain unaltered if this season is included). In the sample, less than 2% of the referee pairs consist of two women. Thus, a binary variable was created that takes the value 1 if the referee pair does not include a woman, and 0 if the team includes at least one woman. The vast majority of referees are white and Spanish.

### Empirical Strategy

We followed previous research on biases among sport referees and used different models to include the main independent variables (nationality/race and gender composition of referee teams) and the rest of control variables (**U**, **V**, and **W**) sequentially (Garicano et al., 2005; Gallo et al., 2013; Pope and Pope, 2015). This entry method allows us to capture any influence of the main independent variables on the number of fouls, and identify the control variables that could moderate this relationship.

To examine the influence of player nationality and gender composition of referee teams, we used the following empirical model:

$$Y_{\text{itg}}={\text{α}}_{0}+{\text{β}}_{1}*RT_{\text{itg}}+{\text{β}}_{2}*Foreign_{\text{itg}}+{\text{β}}_{3}*RT_{\text{itg}}\times Foreign_{\text{itg}}+{\omega \text{U}}_{\text{itg}}+{\theta \text{V}}_{\text{itg}}+{\delta \text{W}}_{\text{itg}}+{\text{ε}}_{\text{itg}}$$

Similarly, to examine the influence of player race and gender composition of referee teams, we used the following model:

$$Y_{\text{itg}}={\text{α}}_{0}+{\text{β}}_{1}*RT_{\text{itg}}+{\text{β}}_{2}*Black_{\text{itg}}+{\text{β}}_{3}*RT_{\text{itg}}\times Black_{\text{itg}}+{\omega \text{U}}_{\text{itg}}+{\theta \text{V}}_{\text{itg}}+{\delta \text{W}}_{\text{itg}}+{\text{ε}}_{\text{itg}}$$

where *i* is a player in season *t* and match *g*, and ε is a random error term.

In both regressions, *Y* is the dependent variable foul rate (in Tables 2, 3) and whether a player received a fifth foul (0/1) (in Table 4). *RT* is a binary variable that captures whether the referee team is all-male (value 1) or mixed (0). In the nationality regression, *Foreign* is a binary variable that takes value 1 if a player is foreign and 0 if she comes from Spain. In the race regression, *Black* is a binary variable that takes value 1 if a player is Black and 0 otherwise. Additionally, the interaction terms between referee team and the player nationality (*RT*_itg_×*Foreign*_itg_) and race (*RT*_itg_×*Black*_itg_) identify if all-male and mixed referee teams call more fouls on foreign or Black players. We used an additional analysis to further examine the influence of race on fouls among foreign players only (the results of this additional analysis do not differ from those described here, and are provided as Supplementary Material).

**TABLE 2:** Regression results (1).

	*Player nationality*				*Player race*			
		
*Variables*	Model 1	Model 2	Model 3	Model 4	Model 1	Model 2	Model 3	Model 4
All-male referee team	−0.0388	−0.0327	−0.0407	0.156	−0.0149	−0.0130	−0.0186	0.177
	(0.0667)	(0.0638)	(0.0630)	(0.203)	(0.0575)	(0.0551)	(0.0549)	(0.202)
Foreign player	−0.266**	−0.181	−0.179	−0.195				
	(0.129)	(0.138)	(0.143)	(0.147)				
All-male referee team ×	0.0916	0.0624	0.0819	0.0979				
Foreign player	(0.101)	(0.0993)	(0.0997)	(0.105)				
Black player					0.0783	0.0778	0.105	0.100
					(0.177)	(0.166)	(0.171)	(0.171)
All-male referee team ×					0.0328	−0.00938	0.00665	0.0161
Black player					(0.139)	(0.137)	(0.139)	(0.138)
Player characteristics		Yes	Yes	Yes		Yes	Yes	Yes
Match characteristics			Yes	Yes			Yes	Yes
Team FE			Yes	Yes			Yes	Yes
Season FE			Yes	Yes			Yes	Yes
Referee FE				Yes				Yes
Constant	4.080***	6.070***	5.961***	5.352***	3.992***	6.067***	5.947***	5.340***
	(0.0879)	(0.287)	(0.379)	(0.662)	(0.0753)	(0.285)	(0.378)	(0.661)
Observations	47,977	47,258	47,258	47,258	47,977	47,258	47,258	47,258
R-squared	0.000	0.027	0.033	0.047	0.000	0.027	0.033	0.047

Influence of nationality and race – Dependent variable: Fouls *40/min played. (a) Robust standard errors in parentheses *** p < 0.01, ** p < 0.05, * p < 0.1. (b) All models are clustered at the player level.

**TABLE 3:** Regression results (2).

	*Player nationality*				*Player race*			
		
*Variables*	Model 1	Model 2	Model 3	Model 4	Model 1	Model 2	Model 3	Model 4
All-male referee team	0.0818	0.0360	−0.0113	−0.403	0.0222	0.0358	−0.0136	−0.341
	(0.203)	(0.195)	(0.203)	(2.267)	(0.181)	(0.175)	(0.182)	(2.266)
Foreign player	−0.477	−0.587*	−0.695**	−0.789**				
	(0.291)	(0.313)	(0.339)	(0.392)				
All-male referee team ×	0.139	0.345	0.345	0.457				
Foreign player	(0.313)	(0.314)	(0.329)	(0.390)				
Black player					−0.335	−0.331	−0.358	−0.413
					(0.347)	(0.367)	(0.382)	(0.427)
All-male referee team ×					0.501	0.544	0.549	0.674
Black player					(0.399)	(0.397)	(0.409)	(0.448)
Player characteristics		Yes	Yes	Yes		Yes	Yes	Yes
Match characteristics			Yes	Yes			Yes	Yes
Team FE			Yes	Yes			Yes	Yes
Season FE			Yes	Yes			Yes	Yes
Referee FE				Yes				Yes
Constant	4.224***	6.230***	6.686***	6.847***	4.157***	6.249***	6.707***	6.801***
	(0.196)	(0.520)	(0.702)	(2.326)	(0.177)	(0.515)	(0.709)	(2.326)
Observations	5,768	5,720	5,720	5,720	5,768	5,720	5,720	5,720
R-squared	0.001	0.040	0.052	0.101	0.000	0.040	0.052	0.101

Influence of nationality and race in one-possession matches – Dependent variable: Fouls *40/min played. (a) Robust standard errors in parentheses *** p < 0.01, ** p < 0.05, * p < 0.1. (b) All models are clustered at the player level.

**TABLE 4:** Regression results (3).

	*Player nationality*				*Player race*			
		
*Variables*	Model 1	Model 2	Model 3	Model 4	Model 1	Model 2	Model 3	Model 4
All-male referee team	0.0150	0.0124	0.0107	0.0452	0.0185	0.0175	0.0167	0.0545
	(0.0190)	(0.0188)	(0.0187)	(0.0578)	(0.0169)	(0.0169)	(0.0169)	(0.0573)
Foreign player	−0.0263	−0.0254	−0.0231	−0.0312				
	(0.0260)	(0.0282)	(0.0285)	(0.0301)				
All-male referee team × Foreign player	0.00453 (0.0296)	0.00913 (0.0293)	0.0105 (0.0293)	0.0213 (0.0312)				
Black player					−0.0104	−0.0118	−0.00283	−0.00673
					(0.0298)	(0.0305)	(0.0301)	(0.0317)
All-male referee team × Black player					−0.00860 (0.0326)	−0.00919 (0.0323)	−0.0102 (0.0321)	−0.00723 (0.0337)
Player characteristics		Yes	Yes	Yes		Yes	Yes	Yes
Match characteristics			Yes	Yes			Yes	Yes
Team FE			Yes	Yes			Yes	Yes
Season FE			Yes	Yes			Yes	Yes
Referee FE				Yes				Yes
Constant	0.255***	0.291***	0.344***	0.713***	0.247***	0.292***	0.342***	0.707***
	(0.0168)	(0.0441)	(0.0566)	(0.194)	(0.0149)	(0.0424)	(0.0555)	(0.193)
Observations	6,697	6,690	6,690	6,690	6,697	6,690	6,690	6,690
R-squared	0.001	0.011	0.025	0.094	0.000	0.011	0.025	0.094

Influence of race and nationality on fifth foul calls – Dependent variable: Fifth foul. (a) Robust standard errors in parentheses *** *p* < 0.01, ** *p* < 0.05, * *p* < 0.1. (b) All models are clustered at the player level.

The remaining factors are identical in both regressions. All variables are listed in Table 1. The vector **U** (Model 2) controls for players’ characteristics. We expect players with better records of points and assists to commit fewer fouls, as teams tend to protect these players from fouling out (Gomez et al., 2016). Different aspects of players might influence the chances of being called for a foul. For example, referees may hesitate to call a foul on a very good player. If a player is a “superstar,” the effect may be even greater (e.g., Deutscher, 2015). We considered superstars players who were drafted in the WNBA and/or play for a national team (further categorizing national teams as top 20, 40, 60, and 80 in the world). We expect fewer foul calls against drafted players and international players from top-ranked national teams. Additionally, we included the position and age of players. Based on previous results, we expect centers and forward who play closer to the basket and secure rebounds to have a higher number of fouls (Sampaio et al., 2006).

The vector **V** (Model 3) controls for characteristics of matches and teams. Binary variables control for derbies, playoff matches, and home court. We expect referees to call more fouls in derbies and playoff matches where rivalries are stronger, outcome uncertainty higher, and prizes at stake bigger (Buraimo et al., 2012; Price et al., 2012). Due to the home bias effect, we also expect referees to call fewer fouls against home players (Dohmen and Sauermann, 2016). Additionally, the vector includes season fixed effects to identify changes over time, and team fixed effects to account for variability in playing style not captured by individual player statistics.

The vector **W** (Model 4) includes individual referee fixed effects to capture unobserved heterogeneity. All models are clustered at the player level to control for individual differences of players. The analysis used linear regression models with robust standard errors (logit and probit models lead to similar results).

In the following, we organize the empirical analysis into three sections; each describes the analysis of different match and foul types:

(1) The first section includes the full sample in a general analysis of fouls in all types of matches. The dependent variable is players’ fouls per match, weighted, following Price and Wolfers (2010), by minutes played. Other types of statistical normalization used in basketball (e.g., per minute or per-36 min statistics), yield similar results.
(2) The second section focuses on one-possession matches. These matches are exciting and end up with a tight result, which increases the stress of both players and referees. For example, Garicano et al. (2005) found a home-team bias in referee decisions in close matches that disappears when the matches are uneven. We define one-possession matches as those in which the final result is a point difference less than four. The dependent variable is again fouls per match (weighted as described above).
(3) The third section focuses on fifth foul calls. The fifth foul is especially important in basketball, because after it the player has to leave the match. Therefore, a subsample was derived, consisting of players who finished the match with either four or five fouls. A binary variable was created that took the value 1 if the player ended the match with five fouls and 0 if she ended with four.

## Results

**FIGURE 1:**
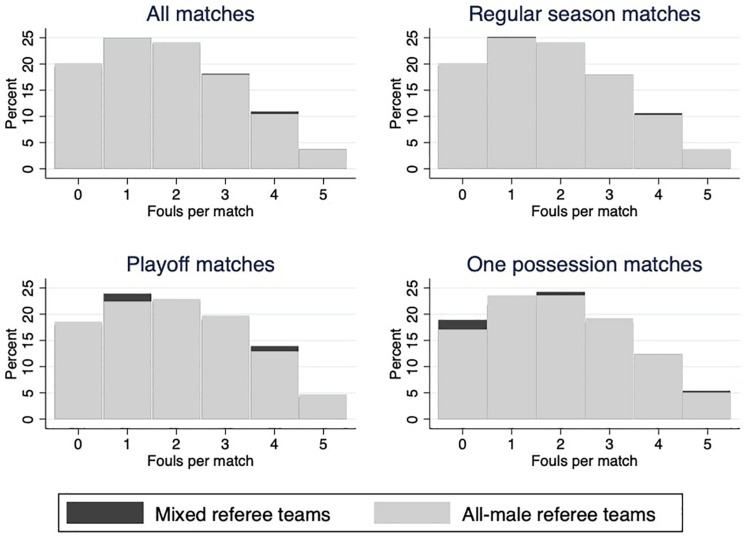
Fouls called (by referee team and type of match).

The first graphical analysis provides an overview of the number of fouls called. Figure 1 displays a histogram that shows the percentage of fouls called on players by both referee teams in different types of matches. Except for small differences, the distribution for mixed and all-male referee teams is very similar.

At an aggregate level, mixed referee teams called an average of 17.54 fouls per match, and all-male referee teams called an average of 17.45. The difference is not significant and shows that the gender composition of the referee teams does not have an impact on the number of fouls in a match. In one-possession matches, we observe a higher number of fouls. On average, both all-male and mixed referee teams called 18.34 fouls per match.

In all regression tables, the models show only the coefficients for the main variables (race, nationality, and gender composition of referee teams), omitting the whole set of control variables. Supplementary Table S1 (Supplementary Material) provides the complete results for the analysis of fouls in all types of matches. The control variables show the expected results, which are consistent and similar throughout the regression models and different analyses (the complete set of results for fouls in one-possession matches and fifth foul calls are available from the authors upon request).

Regarding player characteristics, referees call more fouls on centers. Centers tend to be taller and stronger players who are expected to play near the basket and use their size to the benefit of the team in defensive rebounding and blocking (Sampaio et al., 2006). Players scoring more points and providing more assists receive significantly fewer fouls. The opposite occurs with respect to player turnovers. The number of fouls is also lower for older players and players drafted by the WNBA. However, international players as a whole were not called for more or fewer fouls. Players at home received significantly more fouls than away players, which contradicts the home-team bias effect often found in men’s sports (Dohmen and Sauermann, 2016). Deutscher (2015) found a similar result in the NBA (Deutscher, 2015). Regarding the type of match, the results show that players receive more foul calls in playoff matches.

### Fouls in All Types of Matches

Table 2 reports the influence of nationality and race on the number of fouls per match at the player level. In Model 1 of the regression, we find a significant influence of nationality. The negative sign means that fewer fouls are called on foreign players. However, this result is not consistent: it disappears when we include the variables that control for the player characteristics. Supplementary Table S1 (Supplementary Material) shows how this effect is mainly driven by the influence of older players and players with better records of points and assists. Table 2 provides no evidence that race/ethnicity has an influence on the number of fouls called by the referees.

The gender composition of referee teams does not have a significant effect on the number of fouls. Additionally, the interaction term that tests for biases against foreign or Black players does not yield significant results.

### Fouls in One-Possession Matches

Table 3 reports the regression results for fouls called on players in more stressful matches (i.e., matches in which the difference in the final score is three points or less). A consistent finding throughout the models is that foreign players are called for significantly (*p* < 0.05) fewer fouls in one-possession matches. This finding is in line with previous evidence on referees and stress (Garicano et al., 2005), but does not support theories of in-group discrimination (Tajfel, 1982). Race has no significant influence on the number of fouls in this type of match.

The results also show that the gender composition of referee teams does not play a significant role in the number of fouls called on foreign or Black players in one-possession matches. The interaction term in the regression does not yield significant results either. This means that there is no evidence that all-male and mixed referee teams behave differently toward foreign players.

### Fifth Foul Calls in All Types of Matches

Table 4 includes the regressions where the dependent variable is a special type of foul—the fifth, which brings disqualification. This analysis used only players who ended matches with four or five fouls. The results show that neither nationality nor race has a significant influence on fifth fouls. Neither foreign nor Black players were more likely to be disqualified than their counterparts.

Similar to previous analyses, the results provide no evidence that the gender composition of referee teams influence the number of fifth fouls called. The result is consistent across all models that included different types of fouls and matches.

## Discussion

This paper tests hypotheses about implicit biases. The analysis uses split-second decisions of referees in professional women’s basketball in Spain to examine whether foreign and Black players face a significant bias. The analysis also tests whether the gender composition of referee teams (i.e., all-male vs. mixed teams) has an influence on foul calls. We found two main results: (1) foreign and Black players do not suffer discrimination; and (2) the gender composition of referee teams has no impact on the total number of calls or those called on foreign and Black players. We did, however, observe a bias that favors foreign (but not Black) players in stressful situations, challenging previous evidence on implicit biases and sport referees.

The main results from Table 2 do not support Hypotheses 1 or 2. We found no evidence of the racial/ethnic and nationalistic discrimination found by other researchers among sport referees (Dohmen and Sauermann, 2016) and professionals in other fields (e.g., Jost et al., 2009).

Race is a social construct that has evolved over time (Gannon, 2016) and served societies to create social groups that often result in stereotypes and negative attitudes toward outside members (Park, 1950). In the United States, the historical discrimination against the Black population is rooted in the society and still affects the opportunities of this minority (Deruy, 2016). Such discrimination can be used to explain the implicit own-race bias that research finds among referees in men’s professional sports (Price and Wolfers, 2010; Parsons et al., 2011).

The results from our study suggest that discrimination against players from minorities depends on the context and the country of analysis. Implicit racial associations in sport depend heavily on the historical and cultural evolution of the meaning of race (Kahn, 2013; Suzuki and Von Vacano, 2018). The label “foreigner” also has a strong constructivist component and follow a similar pattern determining the relationships between individuals and the access to social domains (Fox and Miller-Idriss, 2008; Rad and Ginges, 2018).

Previous studies have found biases among referees and judges that favor competitors from the same country at both the individual (Zitzewitz, 2006, 2014; Sandberg, 2018; Krumer et al., 2020) and team level (Mohr and Larsen, 1998; Page and Page, 2010). However, our results show that foreign players do not have more fouls called on them. In fact, we observe the opposite: in stressful situations, foreign players are called for significantly fewer fouls.

This result is in line with the assumption that implicit biases should play a more prominent role when decisions are made under pressure. Previous research finds support for this assumption (e.g., Garicano et al., 2005). The direction of the bias, however, contradicts our initial hypothesis, as implicit bias should favor in-group players (Staats, 2014). One possible explanation relies again on the constructivist process of providing traits with meaning (Suzuki and Von Vacano, 2018), which is likely to differ depending on the context. Consumer ethnocentrism theories illustrate this process.

Nationality and ethnicity have been used to make in-group/out-group distinctions (Tajfel, 1982). In the context of our study, national (Spanish) and white players represent the in-group, and foreign and Black players the out-group. Following the logic of social identity theory, referees will tend to evaluate in-group players unreasonably favorably compared to their out-group counterparts. However, social identity and ethnocentric theories may work in opposite directions. In situations where foreign traits are regarded as better than the national ones, consumers will be motivated to favor national brands, but simultaneously, also comply with the in-group norm that foreign (read *American*) is better (Supphellen and Rittenburg, 2001).

This situation perfectly defines the current status of the Spanish women’s basketball league with respect to other foreign leagues, especially the WNBA in the United States Thus, in the Spanish context, while showing sympathy for national players, referees acknowledge the quality of foreign players, whose higher status can even bias the decisions. This condition also applies to men’s sports. In basketball, previous research provides evidence of the consequences of ethnocentrism in situations of (un)equal perceived quality of foreign and national brands. Berri et al. (2015) found that players born in the United States receive preferential treatment from coaches, who allow them additional time on the court both in the NBA in the United States and Liga Asociación de Clubs de Baloncesto (ACB) in Spain. Our research is the first to show this relationship in women’s sports, although it is significant only in more stressful situations.

In our study, the operational definition of stressful matches—where the difference in the final score is three points or less—may be subject to criticism. Not only does it reduce sample size, but there are other ways to define stress, including crowd size. Several studies explore the relationship between attendance and biases among referees and find significant results (e.g., Garicano et al., 2005; Dohmen, 2008). This is a limitation because we do not have access to attendance data. The basketball federation should consider making this information publicly available not only for research purposes but also for league development plans that encourage participation. Still, we know that women’s basketball has lower attendance than comparable men’s competitions, so that we can expect a smaller influence of the crowd. This could be a reason for the absence of an implicit bias effect in this league, as documented in other professional sports (Dohmen and Sauermann, 2016).

Another limitation of the present study is that we examined only differences between two groups: (1) white vs. Black players, and (2) foreign vs. national players. Potential discrimination that results from the combination of race and nationality is thereby overlooked (Borland and Bruening, 2010). We did perform an additional analysis to rule out this possibility (see Supplementary Material, Supplementary Table S2). We find no evidence that foreign Black players are treated differently than foreign white players. This result suggests that nationality and race as social constructs follow a similar pattern: both lack an association with negative stereotypes in this Spanish sport context.

The third hypothesis does not find support in any type of match and foul call. We found no evidence that the gender composition of referee teams affects foul calls. Neither the total number of fouls in a match nor the fouls called on foreign and Black players differ between all-male and mixed referee teams. This finding is novel in the literature on referee biases in sports. In professional sports, no previous contribution analyzed the relationship between the gender composition of referee teams and foul-call decisions. Women’s sports provide an opportunity to extend knowledge in this line of research.

On the one hand, our results differ from the studies in other fields that find changes in women’s risk behavior when participating in single- vs. mixed-gender groups in experimental settings (Booth et al., 2014; Castillo et al., 2019) and all-male committees with biases against women (De Paola and Scoppa, 2015). On the other hand, our results are consistent with Bagues et al. (2017), who found that the presence of women evaluators in a scientific committee does not have an influence on the quality of selected candidates.

Our findings also support the seminal ideas of Fine (2010), who argued that gender differences in psychological traits will not necessarily have an influence on measurable outcomes. The setting of referees in sports differs from the ones described above. Referees receive specific education and training to be consistent and impartial, but the decisions are time-constrained and do not allow for strategic contemplation. These factors could determine the influence of psychological traits leading to different outcomes.

The study also has limitations in terms of how much light it can shed on the referee gender composition question. Granted, we found no difference in the foul calls made by all-male vs. mixed referee teams. However, the limited number of teams exclusively composed of women is a drawback. We cannot conduct a more informative analysis regarding differences in behavior with presence/absence of individuals from the opposite gender. Similar to previous studies, we found no evidence that the behavior of male referees is affected by the gender of who else is on the team (Castillo et al., 2019), but we cannot rule out the possibility that women referees may behave differently in the absence of men on the team.

We performed a robustness check with the three groups of referees (all-male, mixed, and all-female) and found no significant differences; again, however, the low number of matches with all-female referee teams prevents further analysis. We urge researchers to find suitable settings to perform this analysis. The underrepresentation of women in different roles in women’s sport is a recurrent problem. The governing bodies, federations, and referee committees need to consider strategies to increase the representation of women referees.

Another limitation of this setting is the lack of information on the referees. Unfortunately, we know only the gender and nationality of the referees working in this league. Additional information—such as age, experience, and education—would allow us to identify possible moderating factors. For example, Gallo et al. (2013) found that referees who were born and spent their entire life in the United Kingdom were more likely to penalize foreign, non-white players; however, these authors did not find any significant differences when using purely racial, national, or linguistic criteria. Moreover, it is not possible for us to identify which member of the referee team made the call. That information would be valuable for assessing behavior in the presence/absence of the opposite gender.

Beyond the need for more complete statistics in women’s basketball, the results from this study have other implications for sports organizations. First, the results of this research give league organizers no reason not to assign gender composition of referee teams freely, without fear that players might be treated differently. Second, teams have no reason to exploit referee composition by, for instance, specifically lining up foreign (white or Black) or national players. Third, our findings provide no reason not to use mixed-gender referee teams in *men’s* basketball. The theoretical implications of mixed referee teams calling fouls on male players are different. The empirical setting, however, is almost identical. To advance knowledge of potential cross-gender biases, future research would benefit from the inclusion of female referees in men’s sports. Additionally, such appointments would improve the visibility of women referees in a setting where they are severely underrepresented. Greater visibility could result in greater participation by girls and young women.

The lack of observable biases in women’s basketball in Spain has positive implications, and the good performance of the referees should be acknowledged. Referees undergo specialized education and training to do the job in a consistent and impartial way. Although this is a constant in all sports, referees and judges in some disciplines need to deal with a stronger subjective component (e.g., in dressage; Sandberg, 2018; and figure skating, Zitzewitz, 2006). This is a potential explanation of differences in biases across disciplines.

## Conclusion

Calling a foul on a basketball player is a split-second decision that might be susceptible to bias against members of minority groups. However, we found no evidence of biased decisions with respect to racial/ethnic or national out-groups. In other contexts, negative stereotypes associated with race and foreignness are presumably responsible for the biased decision-making of referees. By contrast, in women’s basketball in Spain, there was no sign of negative bias, and therefore, by extension, no reason to assume negative stereotypes. Indeed, if anything, the results suggest positive stereotypes for foreign players in one-possession matches.

This study also found no evidence that the gender composition of referee teams affects split-second decisions. Across all types of matches, nationalities, and racial/ethnic groups, all-male and mixed referee teams did not differ in number of fouls called. Finally, with respect to all-female referee teams, sports organizations are encouraged to use them more widely, and researchers are encouraged to study them more extensively.

## Data Availability Statement

The datasets generated in this study can be found in online repositories. The names of the repository/repositories and accession number(s) can be found below: https://doi.org/10.7910/DVN/XGDFKI.

## Author Contributions

All authors listed have made a substantial, direct and intellectual contribution to the work, and approved it for publication.

## Conflict of Interest

The authors declare that the research was conducted in the absence of any commercial or financial relationships that could be construed as a potential conflict of interest.

## Footnotes

1 http://ligadia.feb.es and http://www.ligafemenina2.es

## References

[B1] Akerlof G. A. Kranton R. E. (2000). Economics and identity. *Q. J. Econ.* 115 715–753.

[B2] Azmat G. (2019). *Gender Diversity in Teams.* Bonn: IZA World of Labor, 10.15185/izawol.29.v2

[B3] Bagues M. Sylos-Labini M. Zinovyeva N. (2017). Does the gender composition of scientific committees matter? *Am. Econ. Rev.* 107 1207–1238. 10.1257/aer.20151211

[B4] Banaji M. R. Greenwald A. G. (2016). *Blindspot: Hidden Biases of Good People.* New York, NY: Random House.

[B5] Banks R. R. Eberhardt J. L. Ross L. (2006). Discrimination and implicit bias in a racially unequal society. *Calif. Law Rev.* 94 1169–1190. 10.2307/20439061

[B6] Berri D. J. Deutscher C. Galletti A. (2015). Born in the USA: national origin effects on time allocation in US and Spanish professional basketball. *Natl. Inst. Econ. Rev.* 232 R41–R50.

[B7] Bertrand M. Chugh D. Mullainathan S. (2005). Implicit discrimination. *Am. Econ. Rev.* 95 94–98.

[B8] Blair I. V. Dasgupta N. Glaser J. (2015). “Implicit attitudes,” in *APA Handbook of Personality and Social Psychology: Attitudes and Social Cognition*, eds Mikulincer M. Shaver P. R. Borgida E. Bargh J. A. (Washington, DC: American Psychological Association), 665–691.

[B9] Booth A. Cardona-Sosa L. Nolen P. (2014). Gender differences in risk aversion: do single-sex environments affect their development? *J. Econ. Behav. Organ.* 99 126–154. 10.1016/j.jebo.2013.12.017

[B10] Borland J. F. Bruening J. E. (2010). Navigating barriers: a qualitative examination of the under-representation of Black females as head coaches in collegiate basketball. *Sport Manage. Rev.* 13 407–420. 10.1016/j.smr.2010.05.002

[B11] Buraimo B. Simmons R. Maciaszczyk M. (2012). Favoritism and referee bias in European soccer: evidence from the Spanish League and the UEFA Champions League. *Contemp. Econ. Policy* 30 329–343. 10.1111/j.1465-7287.2011.00295.x

[B12] Castillo M. Leo G. Petrie R. (2019). Room composition effects on risk taking by gender. *Exp. Econ* 2019 1–17. 10.4324/9781315269887-1

[B13] Chang E. H. Milkman K. L. (2020). Improving decisions that affect gender equality in the workplace. *Organ. Dyn.* 49 1–7.

[B14] Cohen-Zada D. Krumer A. Rosenboim M. Shapir O. M. (2017a). Choking under pressure and gender: Evidence from professional tennis. *J. Econ. Psychol.* 61 176–190. 10.1016/j.joep.2017.04.005

[B15] Cohen-Zada D. Krumer A. Shtudiner Z. E. (2017b). Psychological momentum and gender. *J. Econ. Behav. Organ.* 135 66–81. 10.1016/j.jebo.2017.01.009

[B16] De Paola M. Scoppa V. (2015). Gender discrimination and evaluators’ gender: evidence from Italian academia. *Economica* 82 162–188. 10.1111/ecca.12107

[B17] Deruy E. (2016). *How Black Lives Matter Activists Plan to Fix Schools.* Available online at: https://www.theatlantic.com/education/archive/2016/08/the-ambitious-education-plan-of-the-black-lives-matter-movement/494711/ (accessed May 24, 2019).

[B18] Deutscher C. (2015). No referee bias in the NBA: new evidence with leagues’ assessment data. *J. Sports Anal.* 1 91–96. 10.3233/jsa-150012

[B19] Dipboye R. L. Colella A. (2013). *Discrimination at Work: The Psychological and Organizational Bases.* New York, NY: Psychology Press.

[B20] Dohmen T. Sauermann J. (2016). Referee bias. *J. Econ. Surv.* 30 679–695. 10.1111/joes.12106

[B21] Dohmen T. J. (2008). The influence of social forces: Evidence from the behavior of football referees. *Econ. Inq.* 46 411–424. 10.1111/j.1465-7295.2007.00112.x

[B22] Fine C. (2010). *Delusions of Gender: How Our Minds, Society, and Neurosexism Create Difference.* New York, NY: Norton.

[B23] FitzGerald C. Hurst S. (2017). Implicit bias in healthcare professionals: a systematic review. *BMC Med. Ethics* 18:19. 10.1186/s12910-017-0179-8 28249596 PMC5333436

[B24] Fox J. E. Miller-Idriss C. (2008). The here and now of everyday nationhood. *Ethnicities* 8 573–576. 10.1177/14687968080080040103

[B25] Gallo E. Grund T. Reade J. (2013). Punishing the foreigner: implicit discrimination in the Premier League based on oppositional identity. *Oxf. Bull. Econ. Stat.* 75 136–156. 10.1111/j.1468-0084.2012.00725.x

[B26] Gannon M. (2016). *Race is a Social Construct.* Available online at: https://www.scientificamerican.com/article/race-is-a-social-construct-scientists-argue/ (accessed March 17, 2019).

[B27] Garicano L. Palacios-Huerta I. Prendergast C. (2005). Favoritism under social pressure. *Rev. Econ. Stat.* 87 208–216. 10.1162/0034653053970267 32495221

[B28] Glaser J. Spencer K. Charbonneau A. (2014). Racial bias and public policy. *Policy Insights Behav. Brain Sci.* 1 88–94. 10.1177/2372732214550403

[B29] Gomez M. A. Ortega E. Jones G. (2016). Investigation of the impact of ‘fouling out’ on teams’ performance in elite basketball. *Int. J. Perform. Anal. Sport.* 16 983–994. 10.1080/24748668.2016.11868943

[B30] Greenwald A. G. Krieger L. H. (2006). Implicit bias: Scientific foundations. *Calif. Law Rev.* 94 945–967. 10.2307/20439056

[B31] Greenwald A. G. McGhee D. E. Schwartz J. L. (1998). Measuring individual differences in implicit cognition: the implicit association test. *J. Pers. Soc. Psychol.* 74 1464–1480. 9654756 10.1037//0022-3514.74.6.1464

[B32] Jost J. T. Rudman L. A. Blair I. V. Carney D. R. Dasgupta N. Glaser J. (2009). The existence of implicit bias is beyond reasonable doubt: A refutation of ideological and methodological objections and executive summary of ten studies that no manager should ignore. *Res. Organ. Behav.* 29 39–69. 10.1016/j.riob.2009.10.001

[B33] Kahn J. (2013). *Race in a Bottle: The Story of Bidil and Racialized Medicine in a PostGenomic Age.* New York, NY: Columbia University Press.

[B34] Kane M. J. Stangl J. M. (1991). Employment patterns of female coaches in men’s athletics: Tokenism and marginalization as reflections of occupational sex-segregation. *J. Sport Soc. Issues* 15 21–41. 10.1177/019372359101500102

[B35] Krumer A. Otto F. Pawlowski T. (2020). *Nationalistic bias among international experts: Evidence from professional ski jumping.* Available online at: https://www.researchgate.net/profile/Alex_Krumer (accessed April 9, 2020).

[B36] Lee W. Cunningham G. B. (2019). Group diversity’s influence on sport teams and organizations: a meta-analytic examination and identification of key moderators. *Eur. Sport Manag. Q.* 19 139–159. 10.1080/16184742.2018.1478440

[B37] Lindner F. (2017). *Choking Under Pressure of Top Performers: Evidence From Biathlon Competitions (No. 2017–24).* Working Papers in Economics and Statistics, University of Innsbruck, innsbruck.

[B38] Mohr P. B. Larsen K. (1998). Ingroup favoritism in umpiring decisions in Australian football. *J. Soc. Psychol.* 138 495–504. 10.1080/00224549809600403

[B39] Monin B. Miller D. T. (2001). Moral credentials and the expression of prejudice. *J. Pers. Soc. Psychol.* 81:33 10.1037/0022-3514.81.1.33 11474723

[B40] Page L. Page K. (2010). *Evidence of Referees’ National Favouritism In Rugby.* *NCER Working Paper Series* 62, National Center for Education Research, Washington, DC.

[B41] Park R. E. (1950). *Race and Culture. Glencoe.* Free Press.

[B42] Parsons C. A. Sulaeman J. Yates M. C. Hamermesh D. S. (2011). Strike three: umpires’ demand for discrimination. *Am. Econ. Rev.* 101 1410–1435. 10.1257/aer.101.4.1410

[B43] Pope B. R. Pope N. G. (2015). Own-nationality bias: evidence from UEFA Champions League football referees. *Econ. Inq.* 53 1292–1304. 10.1111/ecin.12180

[B44] Price J. Remer M. Stone D. F. (2012). Subperfect game: Profitable biases of NBA referees. *J. Econ. Manag. Strategy* 21 271–300. 10.1111/j.1530-9134.2011.00325.x

[B45] Price J. Wolfers J. (2010). Racial discrimination among NBA referees. *Q. J. Econ.* 125 1859–1887. 10.1162/qjec.2010.125.4.1859 32495221

[B46] Rad M. S. Ginges J. (2018). Folk theories of nationality and anti-immigrant attitudes. *Nat. Hum. Behav.* 2 343–347. 10.1038/s41562-018-0334-3 30962601

[B47] Sampaio J. Janeira M. Ibáñez S. Lorenzo A. (2006). Discriminant analysis of game-related statistics between basketball guards, forwards and centres in three professional leagues. *Eur. J. Sport Sci.* 6 173–178. 10.1080/17461390600676200

[B48] Sandberg A. (2018). Competing identities: a field study of in-group bias among professional evaluators. *Econ. J.* 128 2131–2159. 10.1111/ecoj.12513

[B49] Souchon N. Coulomb-Cabagno G. Traclet A. Rascle O. (2004). Referees’ decision making in handball and transgressive behaviors: Influence of stereotypes about gender of players? *Sex Roles* 51 445–453. 10.1023/b:sers.0000049233.28353.f0

[B50] Souchon N. Livingstone A. G. Maio G. R. (2013). The influence of referees’ expertise, gender, motivation, and time constraints on decisional bias against women. *J. Sport Exerc. Psychol.* 35 585–599. 10.1123/jsep.35.6.585 24334320

[B51] Staats C. (2014). *State of the Science: Implicit Bias Review 2014.* Columbus: Kirwan Institute.

[B52] Supphellen M. Rittenburg T. L. (2001). Consumer ethnocentrism when foreign products are better. *Psychol. Mark.* 18 907–927. 10.1002/mar.1035

[B53] Suzuki K. Von Vacano D. A. (2018). *Reconsidering Race: Social Science Perspectives on Racial Categories in the Age of Genomics.* New York, NY: Oxford University Press.

[B54] Tajfel H. (1982). Social psychology of intergroup relations. *Annu. Rev. Psychol.* 33 1–39.

[B55] van Knippenberg D. de Dreu C. K. Homan A. C. (2004). Work group diversity and group performance: an integrative model and research agenda. *J. Appl. Psychol.* 89 1008–1022. 10.1037/0021-9010.89.6.1008 15584838

[B56] Walker N. A. Bopp T. (2011). The underrepresentation of women in the male dominated sport workplace: perspectives of female coaches. *J. Workplace Rights* 15 47–64. 10.2190/wr.15.1.d 22612255

[B57] Wicker P. Breuer C. Von Hanau T. (2012). Gender effects on organizational problems—Evidence from non-profit sports clubs in Germany. *Sex Roles* 66 105–116. 10.1007/s11199-011-0064-8

[B58] Zitzewitz E. (2006). Nationalism in winter sports judging and its lessons for organizational decision making. *J. Econ. Manag. Strategy* 15 67–99. 10.1111/j.1530-9134.2006.00092.x

[B59] Zitzewitz E. (2014). Does transparency reduce favoritism and corruption? Evidence from the reform of figure skating judging. *J. Sports Econ.* 15 3–30. 10.1177/1527002512441479

