# Supplementary material for: Unbiased Decisions Among Women’s Basketball Referees
Source: Front Psychol. 2020 Nov 5;11:566684. doi: 10.3389/fpsyg.2020.566684 (PMC7674594; doi:10.3389/fpsyg.2020.566684)
Supplement: Supplementary file 1 [file Table_1.DOC]

Table S1. Regression results complete (1). Influence of nationality and race – Dependent variable: Fouls *40/minutes played	
	Player Nationality 	Player Race	
Variables	Model 1	Model 2	Model 3	Model 4		Model 1	Model 2	Model 3	Model 4	
										
All-male referee team	-0.0388	-0.0327	-0.0407	0.156		-0.0149	-0.0130	-0.0186	0.177	
	(0.0667)	(0.0638)	(0.0630)	(0.203)		(0.0575)	(0.0551)	(0.0549)	(0.202)	
Foreign player	-0.266**	-0.181	-0.179	-0.195						
	(0.129)	(0.138)	(0.143)	(0.147)						
										
All-male referee team x Foreign player	0.0916
(0.101)	0.0624
(0.0993)	0.0819
(0.0997)	0.0979
(0.105)						
										
Black player						0.0783	0.0778	0.105	0.100	
						(0.177)	(0.166)	(0.171)	(0.171)	
										
All-male referee team x Black player						0.0328
(0.139)	-0.00938
(0.137)	0.00665
(0.139)	0.0161
(0.138)	
										
Player characteristics: 	
Position: Center (omitted)	
Guard		-1.109***	-1.168***	-1.165***			-1.122***	-1.174***	-1.171***	
		(0.181)	(0.173)	(0.173)			(0.180)	(0.172)	(0.172)	
Power forward		-0.439**	-0.487***	-0.486***			-0.436**	-0.481***	-0.481***	
		(0.181)	(0.180)	(0.180)			(0.180)	(0.180)	(0.180)	
Shooting guard		-1.052***	-1.110***	-1.111***			-1.051***	-1.106***	-1.109***	
		(0.185)	(0.181)	(0.181)			(0.185)	(0.181)	(0.181)	
Small forward		-0.783***	-0.821***	-0.819***			-0.786***	-0.820***	-0.819***	
		(0.159)	(0.155)	(0.154)			(0.159)	(0.154)	(0.154)	
Player drafted		-0.268	-0.324*	-0.343*			-0.375*	-0.447**	-0.469**	
		(0.188)	(0.194)	(0.197)			(0.200)	(0.204)	(0.208)	
International player 
(Top 20)		-0.143
(0.159)	-0.205
(0.178)	-0.204
(0.178)			-0.190
(0.152)	-0.242
(0.174)	-0.241
(0.175)	
International player 
(Top 40)		0.142
(0.203)	0.149
(0.215)	0.156
(0.215)			0.0247
(0.194)	0.0452
(0.208)	0.0490
(0.208)	
International player 
(Top 60)		0.0929
(0.211)	0.0954
(0.210)	0.110
(0.211)			-0.0346
(0.212)	-0.0281
(0.215)	-0.0167
(0.216)	
International player 
(Top 80)		0.255 
(0.242)	0.213
(0.257)	0.209
(0.266)			0.162
(0.229)	0.132
(0.246)	0.125
(0.254)	
Player Age		-0.0537***	-0.0679***	-0.0679***			-0.0542***	-0.0682***	-0.0681***	
		(0.00947)	(0.0108)	(0.0108)			(0.00946)	(0.0108)	(0.0108)	
										
Player Productivity: Stats*40/Minutes Played	
Points		-0.0219***	-0.0221***	-0.0219***			-0.0219***	-0.0221***	-0.0218***	
		(0.00631)	(0.00627)	(0.00634)			(0.00635)	(0.00630)	(0.00638)	
Free throws made		0.00849	0.00716	-0.00190			0.00748	0.00616	-0.00295	
		(0.0139)	(0.0140)	(0.0141)			(0.0140)	(0.0141)	(0.0142)	
Free throws missed		0.0231
(0.0153)	0.0225
(0.0151)	0.0207
(0.0151)			0.0233
(0.0153)	0.0224
(0.0151)	0.0206
(0.0151)	
2 point goals made		0.00164
(0.0177)	0.00140
(0.0175)	0.00195
(0.0175)			-0.00123
(0.0181)	-0.00174
(0.0178)	-0.00127
(0.0178)	
2 point goals missed		0.0126
(0.0107)	0.0148
(0.0108)	0.0167
(0.0109)			0.0111
(0.0105)	0.0131
(0.0106)	0.0150
(0.0107)	
3 point goals missed		-0.00757	-0.00809	-0.00735			-0.00707	-0.00761	-0.00688	
		(0.0149)	(0.0145)	(0.0144)			(0.0149)	(0.0145)	(0.0144)	
Offensive rebounds		0.0283*	0.0237	0.0232			0.0268*	0.0221	0.0216	
		(0.0153)	(0.0153)	(0.0153)			(0.0151)	(0.0150)	(0.0151)	
Defensive rebounds		-0.00924	-0.00963	-0.00863			-0.0105	-0.0109	-0.00992	
		(0.0108)	(0.0109)	(0.0109)			(0.0109)	(0.0109)	(0.0109)	
Assists		-0.0502***	-0.0523***	-0.0507***			-0.0489***	-0.0511***	-0.0495***	
		(0.0108)	(0.0112)	(0.0114)			(0.0107)	(0.0111)	(0.0113)	
Steals		-0.00190	-0.00568	-0.00335			-0.00178	-0.00589	-0.00356	
		(0.0148)	(0.0145)	(0.0146)			(0.0147)	(0.0145)	(0.0146)	
Blocks		-0.0349	-0.0360	-0.0313			-0.0382	-0.0394	-0.0349	
		(0.0361)	(0.0361)	(0.0367)			(0.0363)	(0.0364)	(0.0369)	
Turnover		0.107***	0.109***	0.108***			0.107***	0.109***	0.108***	
		(0.0142)	(0.0143)	(0.0143)			(0.0142)	(0.0143)	(0.0143)	
										
Division 1			0.187	0.321*				0.194	0.325*	
			(0.164)	(0.193)				(0.165)	(0.193)	
Playoff match			0.251**	0.368***				0.248**	0.365***	
			(0.100)	(0.120)				(0.100)	(0.120)	
Home team player			0.109***	0.111***				0.111***	0.113***	
			(0.0393)	(0.0388)				(0.0392)	(0.0387)	
Derby match			0.123*	0.0696				0.125*	0.0713	
			(0.0722)	(0.0812)				(0.0723)	(0.0813)	
										
Team FE			Yes	Yes				Yes	Yes	
Season FE			Yes	Yes				Yes	Yes	
Referee FE				Yes					Yes	
										
Constant	4.080***	6.070***	5.961***	5.352***		3.992***	6.067***	5.947***	5.340***	
	(0.0879)	(0.287)	(0.379)	(0.662)		(0.0753)	(0.285)	(0.378)	(0.661)	
										
Observations	47,977	47,258	47,258	47,258		47,977	47,258	47,258	47,258	
R-squared	0.000	0.027	0.033	0.047		0.000	0.027	0.033	0.047	
Note: a) Robust standard errors in parentheses*** p<0.01, ** p<0.05, * p<0.1. b) All models are clustered at the player level	

Table S2. Regression results (1). Influence of race among foreign players only – Dependent variable: Fouls *40/minutes played	
	All matches	One-possession matches	
Variables	Model 1	Model 2	Model 3	Model 4		Model 1	Model 2	Model 3	Model 4	
										
All-male referee team	0.104	0.112	0.105	0.177		0.219	0.477	0.222	-1.671	
	(0.0955)	(0.0963)	(0.0974)	(0.314)		(0.342)	(0.393)	(0.412)	(3.874)	
Foreign black player	0.168	0.208	0.132	0.170		0.0175	0.156	0.00435	-0.0632	
	(0.190)	(0.178)	(0.174)	(0.177)		(0.429)	(0.486)	(0.537)	(0.575)	
										
All-male referee team x Foreign black player	-0.104
(0.152)	-0.145
(0.153)	-0.111
(0.152)	-0.145
(0.152)		0.00481
(0.475)	-0.0716
(0.505)	-0.0407
(0.539)	-0.0249
(0.597)	
										
Player characteristics	Yes	Yes	Yes			Yes	Yes	Yes	
Match characteristics		Yes	Yes				Yes	Yes	
Team FE			Yes	Yes				Yes	Yes	
Season FE			Yes	Yes				Yes	Yes	
Referee FE				Yes					Yes	
										
Constant	3.731***	5.721***	5.282***	3.815***		3.738***	5.146***	5.549***	7.440	
	(0.132)	(0.500)	(0.577)	(1.379)		(0.298)	(0.926)	(1.102)	(6.219)	
										
Observations	13,335	13,333	13,333	13,333		1,637	1,637	1,637	1,637	
R-squared	0.000	0.045	0.061	0.099		0.000	0.087	0.127	0.274	
Note: a) Robust standard errors in parentheses*** p<0.01, ** p<0.05, * p<0.1. b) All models are clustered at the player level	
